# A Plea for the More Frequent Use of the Obstetric Forceps

**Published:** 1881-08

**Authors:** Roger Marvin Griswold

**Affiliations:** North Manchester, Conn.; Member and Fellow of the Connecticut Medical Society, and Delegate to the American Medical Association


ARTICLE II.

### A PLEA FOR THE MORE FREQUENT USE OF THE OBSTETRIC FORCEPS.

ROGER MARVIN GRISWOLD, M. D., North Manchester, Conn.

Member and Fellow of the Connecticut Medical Society, and Delegate to the American Medical Association.

The opposition encountered by Jenner upon the announcement of his discovery was not greater than that which is even now encountered in certain localities, to the more frequent use of the Obstetric Forceps. In the whole history of Medicine, human skill and ingenuity has never devised an instrument or a means for the relief of suffering its equal, and its use deserves not the adverse criticisms which it receives from many of our most intelligent practitioners, but their praise and encouragement for its more frequent use.

Nevertheless, we find men, with an experience extending over a long period of years, men ordinarily accounted successful obstetritioners, resorting but very rarely to their use, and, agreeing that because they have been ordinarily successful, it is best to "let well enough alone." We find also, among many of the younger men in the profession, a class who, relying chiefly upon our standard authors as authorities, accept their statements that the use of this instrument is to be resorted to only in extreme cases, and that multitudinous evils are likely to follow their frequent application. Basing their opinions, as they do, upon the writings of our standard obstetric authors, they are certainly justified in the belief that their frequent use is but an added source of pain and danger both to the mother and child. Churchill enumerates as evils likely to occur, "lacerations of the os-uteri and perineum, perforation by the blades of the uterine or vaginal walls, and bruising, cutting or tearing of the child's head." Cazeaux mentions the same accidents as likely to occur. Elliott speaks of fracture of the skull to the child, and tearing off of the scalp." Bedford mentions as among the possibilities, "fracture of the pelvic bones, laceration of the ligaments and rupture of the uterus." Ramsbotham, in alluding to its dangers, mentions a case in which the *whole uterus was dragged away with the child.*" Meadows estimates the number of deaths of the child in forceps deliveries, as about one in every five, and of the mother as about one in every twenty-nine. Playfair, of all these authors, does not lay particular stress upon the great danger attending their use, but implies rather that the per cent. of still births is decreased in proportion to the frequency of their use.

We are also taught that the proper application of the blades is very difficult, and requires a considerable amount of experience, and a somewhat uncommon skill. Certainly one cannot be *too* skillful in their use, and the more frequently one resorts to them the more skillful will they become, but their proper application *does not* require either a very unusual amount of experience or skill. A clear knowledge of the anatomical relations of the maternal parts to the foetal head, and, above all, a reasonable and moderate degree of confidence in one's own ability, and the faculty of inspiring a like confidence in your patient, are the most needed requisites. Numerous rules and axioms are laid down governing their use and application.

One authority tells us that we must not use them unless the os is fully dilated, another that we may use them if it is partially so. Meigs says " that a man would not be justified in using them within the uterus." Ramsbotham says, " they may be applied when a third part of the margin of the os-uteri can be felt." Lee says, "the head must have escaped from the uterus before the forceps can be used."

Another author says, "they should never be used unless an ear of the child can be felt." Smith says, "One rule is that the head must have rested for six hours on the perineum, and the pains ceased for the same length of time." We are told that we *must not* use them when the soft parts are inflamed, and that when the soft parts commence to be inflamed it is an indication that they should be u sed. We are told that under certain conditions we may use them within the uterus, and that we are never justified in so doing. That we may use them when the os is partially dilated; and that we must never use them unless it is fully dilated.

That they should never be used when pelvic deformity exists, and that they are sometimes of much assistance in such cases. What an array of contradictions, to confuse and dismay the young practitioner, who has on his hands the first case in which they are required. Well may he hesitate, and it is not to be wondered at that so many wait hour after hour, hoping that unaided nature will work out a safe delivery, and are at last obliged to call to their assistance a more experienced or a bolder operator, who soon terminates the case with the forceps. The truth is, it is as impossible to lay down rules which shall govern the use of the forceps, as it would be to enforce a universal law regulating men's diet. It is a lamentable fact that our great authoiities are not always right, but are equally liable to mistakes with the rest of us. And in this matter every physician must be "a law unto himself." There are, and can be, no rules which shall govern their use in all cases. It is ascertained by all authors with which I am acquainted, that the blades must *always* be applied to the bi-parietal diameter of the foetal head. Now it is sometimes an absolute impossibility to determine the exact position of the head, and even if it were always determined, it is often impossible to apply them exactly to this diameter.

I have applied the forceps in almost every possible position of the head, and almost every condition of the uterus and vagina. I have applied them in the uterus with the os fully dilated, and in the uterus when I have had to forcibly dilate the os. I have applied them to the bi-parietal diameter, the occipto-frontal diameter, and twice in face presentations to the occipito-mental diameter. I have applied them in wide pelves, in narrow pelves, and in deformed pelves. I have applied them with an active uterus and with a quiet uterus. Therefore, I say that we must be governed by the circumstances of each separate case, and not by any fixed rule.

As to the dangers attending their intelligent use, a great many of them I deny, and they are all greatly exaggerated, and have their most prolific existence in the fertile brains of their authors. It can be but by the most criminal negligence or ignorance that most of the dangers enumerated as possible, could occur in fact. In the hands of a careful, intelligent man they can be potent for good only. It is neither my place nor my purpose to give instructions regarding their proper application. Suffice it to say that the rules laid down in the text books are no more to be depended upon as infallible regarding the question as to *how* they shall be applied, than they are as to *when* they shall be applied. Circumstances depending upon the position of the head, the style of instrument, &c., must govern each separate case. My object is rather to state some of the annoyances and dangers which a timely application of the forceps will either modify or obviate, and to present a short summary of my experience with them, and first among these I place the shortening of the period of labour, and consequent diminution in amount of pain and exhaustion to the mother. This is very often a matter of great importance, especially in primiperas of a delicate physique and nervous temperament. I am confident that in a large majority of these cases, if a timely use of the forceps were resorted to, we should have much less shock exhaustion, and a consequent better and quicker recovery.

But it is argued by many that if a labor is normal, and there is a chance that nature will effect a delivery, it should be left to her to accomplish.

But the difficulty is, that with our present city and village population, habits of dress and living have developed a race of women, among whom we seldom find a perfectly healthy one, or one of perfect form ; nature is therefore working at a disadvantage, and we seldom have a perfectly normal labor ; surrounding conditions of life have made an aid to nature needful, and suffering women, in this, her greatest trial, reaches her hands to us for help. But many of us, to firmly rooted in the old or well trodden paths, stand indifferently by, and with the means of relief within our easy reach, witness her continued hours of suffering, and the harrowing anxiety of her friends, rather than step out of the beaten track and follow a new fangled notion.

The argument that a labor must be left to nature seems to me as unreasonable as that an abscess should be left to itself to break or a broken bone to unite without surgical aid. In the majority of cases, nature would affect a certain kind of cure in both instances, but the physician who stood by and allowed such a cure to go on might justly be accused of cherishing a relic of barbarism. The vast majority of labors would affect their own deliveries, if left to nature, but many of them do this after hours of suffering which might have been avoided, and consequent exhaustion and depression which would not have ensued, if a timely use of the forceps had been resorted to.

As an illustration, I was called to see a young woman, primipara, ae. 20. Found os fully dilated, and the head half expelled from the womb, but the attending physician informed me that for some reason, although the pains had been quite strong and regular, there had been no apparent progress for the past four hours. The pains were growing weaker, and the woman was exhausted and disheartened. I applied the forceps, and in fifteen minutes delivered her of a fine, lively boy. Had they been applied three hours previous the same happy result might have been attained, and the woman saved those three hours of needless suffering, and the friends and attending physician the same amount of needless anxiety.

A second point to be noticed is the decrease in the number of still births when the forceps are frequently used. It is an undeniable fact that many children are saved by their use, who, were the case left to nature, would perish. Previous to the past few years, before I used the forceps so frequently, but depended upon nature and the occasional assistance of Ergot in tardy labors, my number of still births was in proportion four times as great as now. It is now but rarely the case that when a child is alive at the commencement of labor, it is not alive at delivery. In fact, with the exception of two cases of death in breech presentations, I have not had a single still birth in the past three years, when the labor was at full term, and the child was alive at the beginning of partuition.

As to injuring the child's head it is worse than useless, it is almost criminal, that such a thing should occur, except very rarely. Not one child out of ten that I deliver with forceps has even the marks of the blades upon its head, much less a cut or tear. The great majority of still births (excluding of course those which die previous to labor commencing), are due, not to the *use* of the foreceps, but to the *neglect of their use.* In careful hands it is one of the most harmless instruments ever invented. Except by the greatest carelessness, or grossest ignorance, it cannot cut, tear, or lacerate the child or the mother.

A third important point is the prevention of injury to the soft parts of the mother. Nearly every case of lacerated perineum, versico-vaginal fistulae, &c., would be avoided if the forceps were called into timely use, and used intelligently and with care. With them we have almost complete control of the head, being able to retard and compress it, thus preventing many perineal lacerations, which the strong expulsive pains often cause. Of course lacerated perineums will sometimes occur, but any child that can be delivered naturally without a lacerated perineum, can be so delivered with forceps. Hodge says that the great majority of lacerations and vesico-vaginal fistulae, are due, *not to their use*, but to neglecting to use them.

It is urged by many that their use tends to post-partum hemorrhage. This remains for those who so argue to prove. I do not believe it. The womb ought not, and need not be emptied any more suddenly of its contents in a forceps than in a natural delivery, and if it is not the danger of after bleeding is no greater. It is my custom when using them, as soon as the blades are adjusted, and I am ready to begin delivery, to administer from 20 to 40 drops extract ergot Fl., or if the woman is under an anæsthetic, to direct compression over the womb by an assistant, following it down as its contents are expelled. In this way, I am seldom, or never, troubled with after hemorrhage.

Allow me here to say a word as to the best forceps to use. In the majority of cases, when the head has escaped from the uterus, and is in the inferior strait, or resting on the perineum strait, Thomas' short straight forceps answer every purpose, and are most easy to apply, but when the head is higher up, and for stronger traction and compression we need a long forceps, I am in the habit of using a form manufactured by Codman & Shurtliff, which is a combination of the Simpson blade, the Comstock shaft and the Denman handle. It is light, strong, a splendid tractor and compresser, easy to lock and apply, impossible to injure the woman, and of graceful, elegant appearance, possessing none of the cumbersome, murderous look of many of the other forceps, which is often one reason why so many women and their friends object so strongly to their use.

In conclusion I present a brief summary of my obstetric practice since I have become a convert to the frequent use of the invaluable instruments. It includes 184 cases, extending over a period of three years, including all labors which were over seven months pregnant.

Primiparas 84; multiparas 100 , occipital presentations 161,with 30 forceps deliveries; breech presentations 7 ; two turned, converted into occipital presentations and delivered with forceps ; two body delivered, and delivery completed with forceps, one of those still born ; three natural delivery, one still born ; premature labors 9 ; arm and shoulder presentation 1 ; turned and delivered with forceps 1 ; foot presentation 1; knee and elbow, 1; face 2, one of which delivered with forceps ; tranverse presentation 1 ; turned and delivered with forceps placenta Previa 1 ; still born at full time 2 ; death of mother, none ; I had no lacerated perineums; no vesico-vaginal fistulae; but one case of post-partum hemorrhage ; no inflammation following their use ; no child with cut, bruised or lacerated head ; in fact none of the terrible results we are told follow their use, and yet in 184 cases they were used 37 times or a little less than once in every 5 cases.

